# Comparing observed occurrence of mistreatment during childbirth with women’s self-report: a validation study in Ghana, Guinea and Nigeria

**DOI:** 10.1136/bmjgh-2023-012122

**Published:** 2023-07-21

**Authors:** Hedieh Mehrtash, Meghan A Bohren, Kwame Adu-Bonsaffoh, Theresa Azonima Irinyenikan, Blair O Berger, Ernest Maya, Mamadou Dioulde Balde, Thae Maung Maung, Adeniyi Kolade Aderoba, Özge Tuncalp, Hannah H Leslie

**Affiliations:** 1 UNDP/UNFPA/UNICEF/WHO/World Bank Special Programme of Research, Development and Research Training in Human Reproduction (HRP), Department of Sexual and Reproductive Health and Research, World Health Organization, Geneva, Switzerland; 2 Department of Global Health, University of Washington School of Public Health, Seattle, Washington, USA; 3 Gender and Women's Health Unit, Centre for Health Equity, Melbourne School of Population and Global Health, University of Melbourne, Carlton, VIC, Australia; 4 Department of Obstetrics and Gynaecology, University of Ghana Medical School, Accra, Ghana; 5 Department of Obstetrics and Gynaecology, Faculty of Clinical Sciences, University of Medical Sciences, Ondo, Ondo State, Nigeria; 6 Population, Family and Reproductive Health, Johns Hopkins University Bloomberg School of Public Health, Baltimore, Maryland, USA; 7 Department of Population, Family and Reproductive Health, School of Public Health, University of Ghana, Legon, Ghana; 8 Cellule de Recherche en Sante de la Reproduction en Guinee (CERREGUI), Conakry, Guinea; 9 Department of Medical Research, Ministry of Health and Sports, Yangon, Myanmar; 10 Mother and Child Hospital Akure, Obstetrics and Gynaecology Oke-Aro, Akure, Ondo, Nigeria; 11 Division of Prevention Science, Department of Medicine, University of California, San Francisco, California, USA

**Keywords:** maternal health, public health, cross-sectional survey

## Abstract

**Background:**

There has been substantial progress in developing approaches to measure mistreatment of women during childbirth. However, less is known about the differences in measurement approaches. In this study, we compare measures of mistreatment obtained from the same women using labour observations and community-based surveys in Ghana, Guinea and Nigeria.

**Methods:**

Experiences of mistreatment during childbirth are person-centred quality measures. As such, we assessed individual-level and population-level accuracy of labour observation relative to women’s self-report for different types of mistreatment. We calculated sensitivity, specificity, percent agreement and population-level inflation factor (IF), assessing prevalence of mistreatment in labour observation divided by ‘true’ prevalence in women’s self-report. We report the IF degree of bias as: low (0.75<IF<1.5), moderate (0.50<IF<0.75 or 1.5<IF<2.0) or high (IF≤0.50 or IF≥2.0).

**Results:**

1536 women across Ghana (n=779), Guinea (n=425) and Nigeria (n=332) were included. Most mistreatment items demonstrated better specificity than sensitivity: observation of any physical abuse (44% sensitive, 89% specific), any verbal abuse (61% sensitive, 73% specific) and presence of a labour companion (19% sensitive, 93% specific). Items for stigma (IF 0.16), pain relief requested (IF 0.38), companion present (IF 0.32) and lack of easy access to fluids (IF 0.46) showed high risk of bias, meaning labour observations would substantially underestimate true prevalence. Other items showed low or moderate bias.

**Conclusion:**

Using self-report as the reference standard, labour observations demonstrated moderate-to-high specificity (accurately identifying lack of mistreatment) but low-to-moderate sensitivity (accurately identifying presence of mistreatment) among women. For overall prevalence, either women’s self-report or observations can be used with low-moderate bias for most mistreatment items. However, given the dynamicity, complexity, and limitations in ‘objectivity’, some experiences of mistreatment (stigma, pain relief, labour companionship, easy access to fluids) require measurement via women’s self-report. More work is needed to understand how subjectivity influences how well a measure represents individual’s experiences.

**WHAT IS ALREADY KNOWN?**

- There has been substantial progress in developing approaches to measure mistreatment of women during childbirth.
- However, less is known about the comparability of different measurement approaches, such as labour observations and community surveys with postpartum women.

**WHAT THIS STUDY ADDS**

- Analysis of 1536 labour observations and community survey responses from women in Ghana, Guinea and Nigeria showed that accuracy of labour observations were moderate to high in identifying women who were not mistreated (specificity) and low in identifying those reporting mistreatment (sensitivity) for most mistreatment items.
- Labour observations and community surveys provided comparable prevalence estimates of most types of mistreatment.
- Labour observations underestimated population prevalence of four types of mistreatment relative to community survey: stigma, pain relief request, labour companion present and easy access to fluids.

**HOW THIS STUDY MIGHT AFFECT RESEARCH, PRACTICE OR POLICY**

- Labour observations provide limited accuracy at the individual level for identifying women who will report mistreatment.
- While women’s self-report and labour observations provide comparable prevalence estimates of some types of mistreatment during childbirth, labour observations, a resource-intensive measurement approach, will underestimate certain types of mistreatment: stigma, pain relief request, labour companionship and easy access to fluids.
- More work is needed to understand how subjectivity influences how well a measure represents an individual’s experiences.

## Introduction

Improving women’s experiences of pregnancy and childbirth care includes eliminating mistreatment of women and promoting respectful care for all.^1 2^ The Lancet Global Health Commission on high-quality health systems has also highlighted the importance for all health systems to ‘measure and report what matters most to people’, which includes person-centred care, user experiences, health outcomes, competent care and confidence in the system.^3^

In recent years, there has been a growing effort to quantitatively measure mistreatment during childbirth using different methodological approaches that have produced a wide range of prevalence estimates.^4–7^ To date, researchers have used different sampling techniques, eligibility criteria, definitions, data collection methods (timing, mode, setting) and measurement tools, thus complicating comparison across sites and agreement on the best approach to measure this phenomenon.^4^ The lack of standard measurement may increase the risk of bias (selection, social desirability, information, recall) and complicate cross-study and cross-context comparisons.^4 8^ Understanding relative accuracy of approaches to measure mistreatment and how prevalence based on different measurements can be compared is critical to compare between studies as well as to design and evaluate interventions. Methods for comparing indicator validity across measurement approaches have been developed and applied to measures of health, disease and healthcare quality where a clinical gold standard metric may be available.^9–12^ For patient-reported measures such as mistreatment, the individual is the expert and self-report provides the reference standard.^13^

There have been a few studies conducted in India and Tanzania that have comparatively described the differences between approaches of women’s self-report (community-based postpartum interviews and facility-exit interviews) and labour observations of how women are treated during childbirth.^14–19^ In this study, we compare measures of mistreatment obtained through labour observation with a community-based postpartum survey of women in Ghana, Guinea and Nigeria to assess the individual-level and population-level accuracy of mistreatment measurement.

## Methods

### Patient and public involvement

A technical consultation with representatives from advocacy groups, non-governmental organisations, research organisations, universities, professional associations and United Nations agencies was held at the WHO in November 2013 and informed the research questions and design of survey instruments in the WHO ‘How women are treated during facility-based childbirth’, study from which data for these analyses were obtained.^4 20^ Women who had recently given birth were involved in exploratory formative research informing tool development, content validity testing and providing feedback on the validity testing of the tools.^21^

### Study design and participation

This is a secondary analysis of data from the WHO study: ‘How women are treated during facility-based childbirth’, designed to develop and validate two tools (labour observation tool and community survey tool) to measure the mistreatment of women during childbirth in health facilities in Ghana, Guinea, Nigeria and Myanmar. The protocol for the formative phase and methodological development of these tools is available,^21 22^ and the results of the primary analysis have been published.^4^ An author reflexivity statement reflecting on the dynamics of our international partnership is available in online supplemental file 1.

10.1136/bmjgh-2023-012122.supp1 Supplementary data

Briefly, in each country, three facilities were purposively selected based on the following inclusion criteria^1^: facilities not included in the formative phase of tool development^2^; secondary-level facility or higher^3^; ≥200 births per month; and^4^ well-defined community-catchment area. Women were eligible for the survey if they were admitted for childbirth at a selected facility, were ≥15 years old, were residents of the facility catchment area and were able to and did provide consent. Exclusion criteria included close relationship with facility employees, women who were distressed or otherwise unable to reasonably provide consent or insufficient contact information for follow-up.

The labour observations were continuous, one-to-one observations of women by non-clinical study researchers from admission, throughout labour and childbirth, until 2 hours post partum. For the community-based survey, women were initially recruited at the facility they gave birth in, shared contact information and were contacted starting 2–3 weeks after childbirth to schedule the survey. Community surveys were conducted in a private location chosen by the participant at up to 8 weeks post partum, and were administered by an interviewer using a tablet-based data collection form. All data collectors were women aged ≥18 years, who were trained in research methods, and not health workers, clinical trainees or students. Training covered each item in the observation checklist, with brief vignettes provided as illustration whenever possible (see online supplemental file 1 for further details).

### Sample size

A total of 2016 women participated in the labour observations and 2041 women participated in the community surveys across Ghana, Guinea and Nigeria (no labour observations were conducted in Myanmar; therefore data from Myanmar were not included in this analysis). Women eligible for participation in both the labour observations and community surveys were able to be linked via the unique medical record numbers assigned by the health facilities. In total, 1536 women (76.2%) participated in both the community survey and labour observations and were eligible for this analysis: 779 women in Ghana (50.7%), 425 women in Guinea (27.7%) and 332 women in Nigeria (21.6%).

### Measures

For comparative purposes, the items measuring specific types of mistreatment were based on matching the same questions in both the labour observation and community survey tool for the following domains^1^: physical abuse, verbal abuse, stigma and/or discrimination, failure to meet professional standards of care and poor rapport between women and providers.

Across physical and verbal abuse domains, similar items were categorised. We grouped physical abuse items in two categories: (1) any slap, punch, hit or pinch; and (2) any forceful downward abdominal pressure. The verbal abuse items were also grouped in two categories: (1) any shout, scold, insult or negative comments (about the woman’s appearance or her sexual activity, or about her baby); and (2) any threatened outcome of care (medical procedure, physical violence, poor baby outcome, withholding care), or blame. All other physical abuse items (any kick, tie to bed, held to bed) and verbal abuse items (any hiss) were excluded for the purposes of this analysis due to very low incidence in the primary analysis.^4^ The stigma and/or discrimination variable was developed as a composite of the following items: women’s receipt of negative comments about their ethnicity/race, religion, age, marital status, education/literacy or economic status.

Failure to meet professional standards of care items included: (1) non-consented care for caesarean section, (2) non-consented care for episiotomy, (3) non-consented vaginal examination (4) disclosure of private information about vaginal examinations, (5) pain relief requested, (6) pain relief not received and (7) no staff member present during childbirth (pain relief and staff presence items among women with vaginal births only).

Items on ‘poor rapport among women and providers’ consisted of two categories: (1) labour companionship; and (2) mobilisation during labour and easy access to water or oral fluids during labour (both among women with vaginal births only).

### Analysis

We applied validation approaches developed for health and healthcare quality indicators.^9^ Women’s self-reported experiences of mistreatment during childbirth were used as the reference standard against which labour observations were assessed. For self-reported (reference) and externally observed measures of mistreatment 2×2 tables were constructed (table 1). Missing and do not know responses were excluded from the 2×2 tables. We provide definitions for validation metrics applicable to self-reported surveys treated as the reference standard at both the individual level (sensitivity, specificity and percent agreement) and the population level (inflation factor) (table 2).

**Table 1:** 2×2 table depicting women’s self-report (reference standard) and observed mistreatment^9^

	Reference standard		
Comparison	Self-reported mistreatment	Self-reported no mistreatment	Total
Observed mistreatment	A	B	A+B
Observed no mistreatment	C	D	C+D
Total	A+C	B+D	N (A+B+C+D)

**Table 2:** Validation metrics applied when self-reported measures provide the reference standard (adapted from^9^)

Term	Definition	Formula
Sensitivity	The proportion of individuals who truly were mistreated (reported mistreatment) who were classified as mistreated by observation.	A/(A+C)
Specificity	The proportion of individuals who truly were not mistreated (reported no mistreatment) who were classified as not mistreated by observation.	D/(B+D)
Percent agreement	The proportion of agreement between observation and self-report among the total sample.	(A+D)/(N)
Inflation factor	Ratio of observed mistreatment to true (self-reported) mistreatment.	(A+B)/(A+C)

Sensitivity and specificity provide accuracy of identifying true positives and true negatives, respectively, at the individual-level. Percent agreement is an indication of accuracy at the individual-level that is also affected by prevalence of the item. Inflation factor (IF) provides a metric of population-level accuracy, the ratio of prevalence based on imperfect measurement to the truth.^9^ Measures may be inaccurate at the individual-level but relatively accurate at the population-level if false positives and false negatives roughly balance out.^9^ In our analysis, the IF reflects the prevalence of specific mistreatment items as it would be in the observation only (Pr=(A+B)/N) divided by the true prevalence in the women’s self-report (P=(A+C)/N). We calculated each metric for each type of mistreatment across domains. We classified bias in IF as low (0.75<IF<1.5), moderate (0.50<IF<0.75 or 1.5<IF<2.0) or high (IF≤0.50 or IF≥2.0).^11^ While we calculated IF directly from study data for these settings, it can be extrapolated to settings with different true prevalence of mistreatment by calculating observed prevalence using the formula Pr=P(Sens*+*Spec-1)*+*(1-Spec).^9^

We calculated country-specific percent agreement and IF to explore accuracy across each study setting. We then plotted true prevalence (self-report) compared with observed prevalence across the full range of possible prevalence to illustrate the difference in IF as prevalence of mistreatment varies.^9^ We selected items for illustration representing the domains of mistreatment assessed and for a range of true prevalence and IF levels.

## Results

Table 3 presents the socio-demographic and obstetrical characteristics of the analytical sample. Nearly half of women were 20–29 years of age (756/1536, 49.2%). Most women were currently married or cohabitating (88.5%, 1360/1536), and about half of women had no or primary education (no education: 227/1536, 14.8%; some primary education: 156/1536, 10.2%, complete primary education: 410/1536, 26.7%). Most women had a vaginal birth (1356/1536, 88.3%). Distribution of socio-demographic and obstetrical characteristics of the women who were linked between the two tools were generally comparable to women who were not linked, with some small differences in age, marital status, parity and mode of birth (p<0.05) (online supplemental table 1).

**Table 3:** Socio-demographic and obstetrical characteristics (N=1536)

	Total N (%)
Country	
Ghana	779 (27.7)
Guinea	425 (50.7)
Nigeria	332 (21.6)
Age (years)	
15–19	196 (12.8)
20–29	756 (49.2)
≥30	584 (38.0)
Marital status	
Single, divorced or widowed	174 (11.3)
Married or cohabitating	1360 (88.5)
Unknown	2 (0.1)
Highest level of education	
No education	227 (14.8)
Some primary education	156 (10.2)
Complete primary education	410 (26.7)
Complete secondary education	440 (28.7)
Complete post-secondary education	262 (17.1)
Vocational, other or unknown	41 (2.7)
Parity	
1 (first birth)	952 (61.9)
≥2 births	584 (38.0)
Mode of birth	
Vaginal	1356 (88.3)
Caesarean section	179 (11.7)
Do not know	1 (0.1)
Woman’s birth position	
Dorsal/supine	952 (62.0)
Lithotomy	519 (33.8)
Squatting	4 (0.3)
Sitting	1 (0.1)
Lying on her side	6 (0.4)
Other/unknown	53 (3.5)

Table 4 reports measurement agreement for each type of mistreatment as a domain (eg, physical abuse) and by item grouping (eg, slap, pinch, punch, hit). Overall, the percent agreement ranged from 50% (non-consented episiotomy) to 96.5% (any stigma or discrimination). The following sections present results by domain (physical abuse, verbal abuse, stigma or discrimination, non-consented care, breaches of confidentiality, pain relief, supportive care and autonomy) and item in detail.

**Table 4:** Comparison of self-reported and observed mistreatment (N=1536)

	A: Reported, observed	B: Not reported, observed	C: Reported not observed	D: Not reported, not observed	Sensitivity (%, 95% CI) (A/(A+C))	Specificity (%, 95% CI) (D/(B+D))	% Agreement (A+D)/(N))	Inflation factor (IF) (A+B)/(A+C)	Risk of bias*
Physical and verbal abuse									
Any physical abuse (N=1536)	83	142	107	1204	44 (41 to 46)	89 (88 to 91)	84	1.18	Low
Any slap, pinch, punch, hit (N=1378)	46	102	46	1204	50 (47 to 53)	92 (91 to 94)	89	1.61	Moderate
Any forceful downward pressure on abdomen (N=1278)	22	12	40	1204	35 (33 to 38)	99 (99 to 100)	96	0.55	Moderate
Any verbal abuse (N=1536)	334	269	215	718	61 (58 to 63)	73 (71 to 75)	68.5	1.10	Low
Any shout, scold, insult, mock, negative comments‡ (N=1435)	292	259	166	718	64 (61 to 66)	73 (71 to 76)	70	1.20	Low
Any threats or blame† (N=815)	42	10	45	718	48 (46 to 51)	99 (98 to 99)	93	0.60	Moderate
Stigma or discimination									
Any stigma or discrimination (N=1536)	2	7	55	1472	4 (3-4)	100 (99 to 100)	96	0.16	High
Failure to meet professional standards of care									
Non-consented care among procedures									
Caesarean section (N=144)	6	20	14	104	30 (28 to 32)	84 (82 to 86)	76	1.30	Low
Episiotomy§ (N=105)	44	8	32	21	58 (56 to 61)	72 (70 to 75)	62	0.68	Moderate
Vaginal examinations (N=986)	377	144	230	235	62 (60 to 64)	62 (60 to 64)	62	0.86	Low
Vaginal examinations— private health information disclosed during examination (N=1057)	69	67	115	815	38 (36 to 39)	92 (91 to 94)	83	0.74	Moderate
Pain relief requested§	50	58	231	1186	18 (16 to 20)	95 (94 to 96)	81	0.38	High
Not received (n=50)	15	4	4	25	79 (77 to 81)	86 (84 to 88)	83	1.00	Low
No staff member present when baby born§ (n=1519)	10	54	24	1431	29 (27 to 31)	96 (95 to 97)	95	1.88	Moderate
Poor rapport between women and providers									
Supportive care: Woman had a companion present during labour and childbirth (N=1518)	107	70	444	897	19 (17 to 21)	93 (91 to 94)	66	0.32	High
Autonomy									
Woman did not have easy access to water or oral fluids during labour§ (N=1312)	150	92	371	686	29 (24 to 34)	88 (85 to 91)	64	0.46	High
Woman not told she could and did not mobilise during labour (N=1522)	840	165	158	359	84 (82 to 86)	69 (66 to 71)	78	1.01	Low

*Risk of bias: IF as low (0.75<IF<1.5), moderate (0.50<IF<0.75 or 1.5<IF<2.0) or high (IF≤0.50 or IF≥2.0).

†Threats of medical procedure, physical violence, poor baby outcome, withholding care.

‡Negative comments regarding the woman’s or baby’s appearance, or mother’s sexual activity.

§Among women with vaginal births only.

### Physical and verbal abuse

Indicators of physical and verbal abuse were generally assessed with higher specificity than sensitivity. This means that observers could accurately identify women not experiencing mistreatment in most cases (specificity ranging from 73% for any verbal abuse to >99% for forceful downward pressure, any threats), but showed lower accuracy in identifying those experiencing mistreatment (sensitivity ranging from 35% for forceful downward pressure to 64% for any scold, shout, etc). Percent agreement ranged from moderate for any verbal abuse (68%) to high (96%) for comparatively less common types of mistreatment, such as forceful downward pressure.

At the population-level, three items demonstrated low bias, with observation slightly overestimating prevalence relative to self-report: any physical abuse, any verbal abuse and any shouting, scolding, etc. Population-level bias was moderate for any slap, pinch, punch or hit (IF 1.61, observed more often than reported), forceful downward pressure on abdomen (IF 0.55, observed less than reported) and any threat (IF 0.60, observed less than reported).

### Stigma

Accuracy of stigma measurement should be interpreted with caution due to low case counts (n=2 both observed and reported, n=7 observed but not reported, n=55 reported but not observed). We found low sensitivity (4%) and perfect specificity for labour observations (100%). IF indicated high risk of bias due to under-observation of reported incidents of stigma.

### Failure to meet professional standards of care

Items related to failure to meet professional standards of care were measured with variable sensitivity (ranging from 18% for pain relief requested to 62% for non-consented vaginal examination and 79% for pain relief not received, noting small cell counts for ‘pain relief requested but not received’) and moderate-to-high specificity (from 62% for non-consented vaginal examination to 96% for no staff member present when baby born). Percent agreement was higher for less common mistreatment items, such as disclosure of private health information or no staff member present when baby born (83% and 95%) than for more common mistreatment items like non-consented vaginal examinations (62% agreement). IF indicated low risk of bias at the population level for estimation of non-consented caesarean section (IF 1.3), non-consented vaginal examinations (IF 0.86) and not receiving pain relief (IF 1.0). IF indicated moderate risk of underestimation using observations to measure non-consented episiotomy (IF 0.68) and disclosure of private health information (IF 0.74) and high risk of underestimation using observations for pain relief requested (IF 0.38). IFs showed moderate risk of overestimation using observations for no staff member present (IF 1.88).

### Poor rapport between women and providers

Two items related to poor rapport demonstrated low sensitivity and high specificity on observation: companion present during labour and birth and no easy access to fluids. In contrast, the item on whether the woman was told to and did mobilise during labour was sensitive (84%) and moderately specific (69%) on observation. The mobilisation item also showed good percent agreement (79%) and population-level accuracy (IF 1.01). Presence of a companion and ease of access to fluids showed lower percent agreement (66% and 64%) and high risk of underestimation at the population-level, suggesting that observations are not likely to accurately identify presence of a labour companion or easy access to fluids at a population-level.

### Estimating inflation factors across true prevalence

Across the country-specific analysis, most mistreatment items that performed well in the full sample (low IF, high percent agreement) also demonstrated individual and population-level accuracy within each country (online supplemental table 2). An exception is mobilisation during labour, which was much more common in Ghana and Nigeria than in Guinea (over 85% vs 8.5%); despite good percent agreement in all countries, IF was very high in Guinea due to observers reporting higher observed prevalence relative to the very low prevalence reported by women. Across items that did not perform well in the full sample (ease of access to fluids, labour companionship), measurement properties were adequate in at least one country.

**Figure 1:**
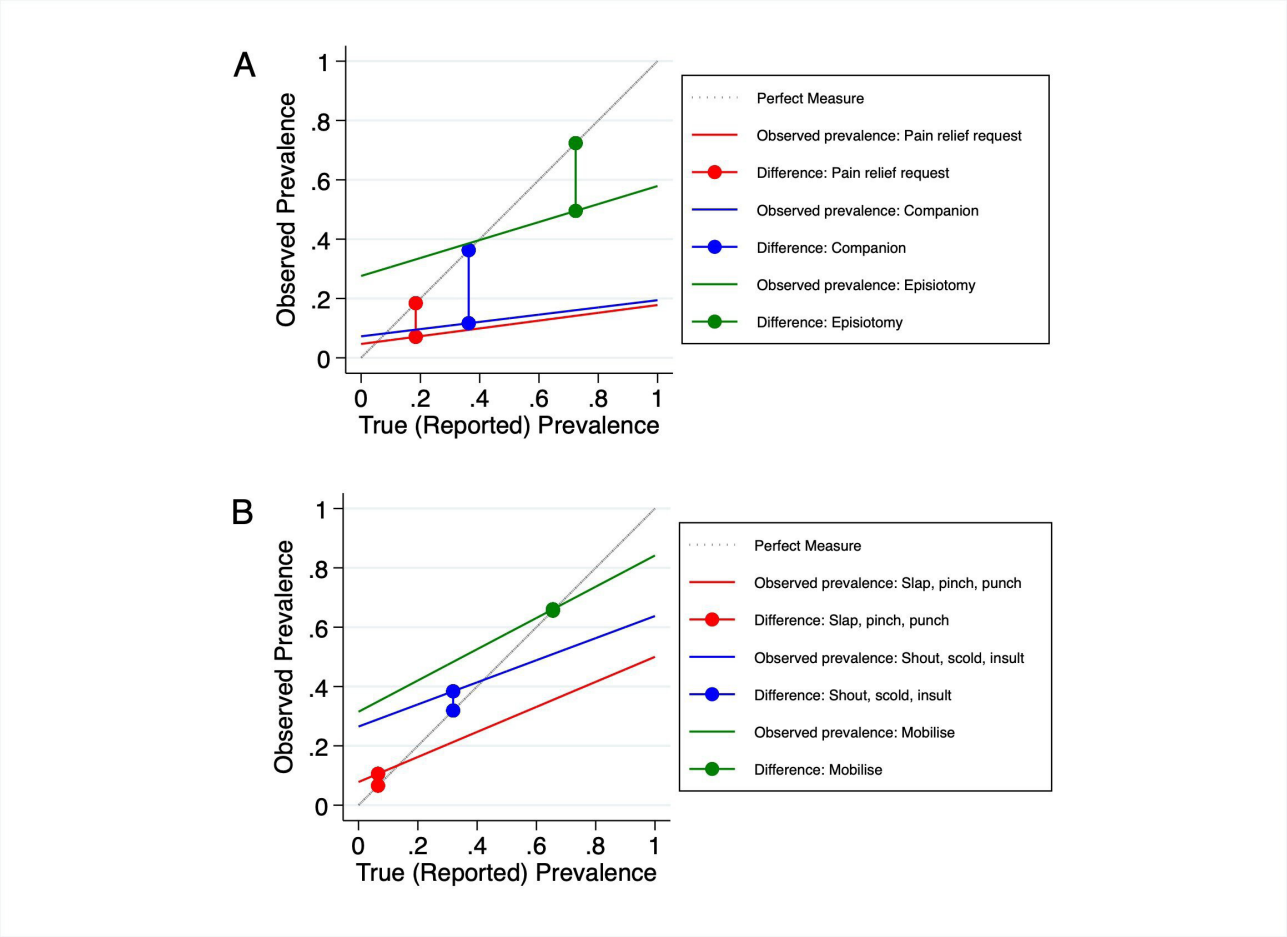
Estimating inflation factors across true prevalence**Panels A and B show the differences between ‘perfect measure’ (100% agreement between a woman’s self-report and observations of mistreatment items) and observed measure in the study. Panel A illustrates with IF <1 (observed less than reported) and Panel B illustrates items with IF > 1 (observed greater than reported). The gaps between true prevalence (perfect measure of prevalence) and observed prevalence in the current study are indicated by coloured dots.

Figure 1 extrapolates beyond the current study by depicting the prevalence of mistreatment based on observation across a range of true (self-reported) prevalence levels, highlighting the differences found in our analyses. Panel A includes items with IF<1 (observed less than reported in our study) and low (pain relief), moderate (low presence of companion) and high (non-consented episiotomy) prevalence. Observation would likely underestimate true prevalence across most possible prevalence levels for whether the woman requested pain relief or had the presence of a labour companion; observation would overestimate true prevalence of non-consented episiotomy in low or moderate prevalence settings (<40%).

Panel B includes items with IF>1 (observed greater than reported in our study) and low (slap, pinch, punch, hit), moderate (shout, scold, insult, mock) and high (woman told she could and did mobilise) prevalence. The differences between observed and reported tended to be less for items with IF>1 both in our study and for most possible prevalence values. This suggests that for items where observers overestimated prevalence in our study, the difference between observed and women’s self-reported experiences of these types of mistreatment is likely to be small across most true prevalence levels, and only indicative of high bias in settings of very high or very low prevalence, as noted above for high IF for women not told she could and did mobilise in the low prevalence setting of Guinea.

## Discussion

This study uses a novel application of validation methods to quantify the accuracy of labour observations relative to women’s self-report (reference standard) when measuring mistreatment during childbirth. Overall, labour observations provided more specificity than sensitivity, meaning this measurement approach may be useful to accurately identify those not experiencing mistreatment. Accuracy of labour observation in identifying women truly experiencing mistreatment was lower (sensitivity) for many items. At the population-level, most observation items could be used to estimate prevalence of mistreatment with low or moderate risk of bias, relative to women’s self-report. Our findings suggest that labour observations can be used at the individual level to screen out items within the abuse domains of mistreatment (high specificity) and to estimate population prevalence of overall physical and verbal abuse with low bias. However, observations of stigma, pain relief request, presence of a companion and easy access to fluids showed high risk of bias, meaning labour observations would likely underestimate true prevalence. Our analyses show challenges of using labour observation in comparison to the self-reported women’s experiences of mistreatment during childbirth.

Our analyses of the two measurement approaches provide insight on the subjectivity of women-related experiences of care measures. For example, we found smaller differences between observation and self-report for more objective measures of types of verbal abuse (eg, any shout, scold, insult), but larger differences for more subjective types of mistreatment (eg, threatening to withhold treatment). These results were similar to another study conducted in Tanzania, which found that observers mostly recorded shouting and scolding.^18^ Similarly, within types of physical abuse, forceful downward pressure on the abdomen was also reported more commonly than it was observed, meaning that observations are likely to underestimate this type of mistreatment. A recent systematic review found that the prevalence of fundal pressure during second stage labour was 23.2% globally,^23^ despite fundal pressure not being recommended by WHO.^24^ This highlights the importance of de-implementing such harmful practices, and how de-implementation of non-evidence-based practices may influence women’s experiences of care.^25^ Another example is women’s experiences of stigma and discrimination—our analysis showed that labour observations are not likely to accurately identify stigma at either an individual-level or population-level. Stigma is an inherently subjective measure that reflects women’s lived experiences, personal and social identities and expectations of the health system that an observer may not be able to discern. As such, for these items, it is not surprising that observations underestimate women’s experiences of stigma, and our analyses show what is likely missed in the absence of asking women about their experiences of care. Future research to understand the mechanisms driving stigma and discrimination during childbirth in various contexts is needed.^26^

Our findings also highlight the importance of focusing on women’s experiences of care that are often shaped by individual expectations of childbirth. Observations showed low sensitivity and high risk of bias in measuring lack of easy access to fluids and lack of a labour companionship. Labour companionship and ease of access to fluids are recommended by WHO for all women throughout labour and birth,^27 28^ and have important clinical and interpersonal benefits for both the woman and baby, including reducing mistreatment during childbirth.^29^ Despite being recommended by WHO, there are substantial gaps between the recommendations and ensuring all women have access to support from a labour companion and easy access to fluids. For example, a recent global scoping review found wide variation in coverage of a labour companionship, with one-third of the studies reporting that coverage of a labour companionship for <40% of women.^30^ Our findings on relative inaccuracy of observation for these items might result from how labour companionship and ease of access fluids are interpreted as observers are more shaped by these system-level and facility-level policies while women report their direct experiences with the health system. Furthermore, how these items are implemented across different contexts should be addressed by adoption of standard policies and infrastructure to support women’s choices of a labour companionship.^27 31^

There have been various quantitative methods of measuring mistreatment during childbirth. While measuring mistreatment using either a community survey or labour observation depends on resources available, we also note that any research or monitoring related to women’s experiences of care should allow women to report on what matters most to them. Shortened and full versions of the tools used in our analyses can inform key areas of focus, while cognisant of certain types of mistreatment that may be feasible to rule out but difficult to detect with observation and might need to be explored further using two methods.^32 33^ Furthermore, the measurement approaches need to be interpreted in the context of tool implementation for effective action on improving quality of care. There are also practical and logistical differences between the methods: community surveys (possible recruitment from facility, travel to communities, follow-up), labour observations (resource intensive, potentially 24 hours per day as in our study, challenging in some settings) and exit interviews (recruitment and survey post partum at the facility, risk of social desirability bias when conducted on facility grounds). Differences in women’s recall and understanding of mistreatment between exit interviews and subsequent surveys may also have implications for validity metrics presented here.^9 13^ More recently, more evidence has been emerging using phones to conduct surveys to capture experience of care during the postnatal period.^34 35^ Most validation studies on quality of maternal and newborn care have used observation as a reference standard^10–12^ including a recent study on mistreatment measures.^19^ We used women’s self-reported measures as the reference standard in this study in keeping with mistreatment measures as person-centred process quality measures emphasising women’s voices, preferences and needs.^13^ Focusing on women’s reported experiences using the community survey may be an effective approach in measuring certain nuances of mistreatment highlighted in this study, while not compromising women’s experiences that may be limited using facility-exit interviews (due to both bias around collecting information about mistreatment while still in a facility environment, as well as insufficient time for a woman to process her birth experience).

Our analysis has both strengths and limitations. This study builds on the strengths of the WHO ‘How women are treated during facility-based childbirth’ study, which developed tools specifically to capture mistreatment that have been validated in four countries.^32 33^ The robust approach to the analysis allowed us to consider individual-level and population-level accuracy of labour observations relative to the community survey. This was a cross-sectional study conducted across public facilities in urban areas, and not all participants had complete data for inclusion in this analysis; therefore, the patterns and types of mistreatment may not be generalised to the population across these three countries. The sample size for certain subitems of mistreatment was small (<5 counts per cell) and therefore may limit our interpretation of the differences between the two tools. As the surveys were conducted up to 4–8 weeks post partum, recall bias is a possibility and a woman may have under-reported mistreatment given that her childbirth experience may be influenced by factors such as her child’s current health status. IF estimates are based on the prevalence of mistreatment in community surveys in this study; these estimates would change in settings of higher or lower prevalence for each type of mistreatment. The original study was a novel attempt at measuring mistreatment across a community survey and labour observations. Despite efforts to standardise items across observers (online supplemental file), some items (eg, ease of access to fluids or labour companionship) were more difficult to define equivalently than others (eg, verbal or physical abuse). Inconsistency between observers might affect the accuracy of observed behaviours compared with a woman’s self-report.

### Implications for future research

Future measurement studies should ensure usage of standardised measures and tools. First, researchers should assess how well the measure represents the probable range of peoples’ experiences (content validity). Validation should occur in the context where the research is taking place, considering both the type of care (eg, primary care or facility) and geographical or cultural context. Second, researchers should consider how a measure performs across populations. Not all measures need to be reliable across populations and settings, but this is an important consideration when considering the generalisability of findings. Using existing measures is key to ensure reliability across settings or populations. Finally, explicit definitions for items can be incorporated and tested in study data collection training (via vignettes, inter-rater reliability) to ensure accuracy and comparability against self-report.

While measuring women’s experiences during labour and childbirth is important, it is equally essential to consider the need to measure and intervene along the continuum of maternal health including antenatal, postnatal and abortion care. Given that antenatal care is the first interaction of a woman with the maternal health system in many cases, poor experiences of care at this time point may influence their future choice of facility or provider, or in absence of options, ultimately influence their preferred place of birth. More work is needed to develop approaches and measures to better understand how women experience the continuum of maternity care, particularly at the antenatal and postnatal care time points.

Developing a comprehensive set of validated measures covering both the provision and experiences of care is critical to address quality of care, women’s rights and improve health outcomes. More work is needed to develop implementation models to provide improved person-centred maternity care, building on existing recommended (but under-implemented) interventions such as labour companionship and improved communication and approaches to truly informed consent.^24^ Quality improvement programmes and routine health management information systems likewise should measure and monitor user experiences of care, as current quality of care assessments are insufficient to implement effective person-centred care programmes. Implementation of WHO recommendations on intrapartum care and the WHO Labour Care Guide may help to facilitate that respectful maternity care is provided and routinely embedded in maternity care and facility records.^36 37^ Adaptation of WHO recommendations of the intrapartum care guidance in local contexts is essential for effective implementation of women’s receipt of high quality, respectful maternity care.^37 38^

## Conclusion

Our analysis shows that labour observations measure most types of mistreatment during childbirth but provide limited accuracy at the individual level for identifying women who will report mistreatment. Important types of mistreatment, such as stigma, pain relief request, presence of a companion and easy access to fluids, showed high bias when measured through observation in these settings, meaning that observations are likely to underestimate true prevalence. For person-centred measures, such as mistreatment, the reference standard is an individual’s self-reporting. More work is needed to understand how subjectivity influences how well a measure represents an individual’s experiences. As measurement of mistreatment advances, careful use and interpretation of available tools for appropriate individual and population-level inference remains critical.

## Data Availability

Data are available upon reasonable request. The analytical study data set from the ‘WHO Study: How women are treated during facility-based childbirth’ is de-identified and archived through WHO/HRP’s electronic record management system. Data requests with an expression of interest in pursuing multicountry secondary analyses with a specific research question can be made to srhmph@who.int. More information about the study tools are available here: https://bmcmedresmethodol.biomedcentral.com/articles/10.1186/s12874-018-0603-x. More information about the primary publication from the study here: https://www.thelancet.com/journals/lancet/article/PIIS0140-6736(19)31992-0/fulltext.
