# Supplementary material for: Comparing observed occurrence of mistreatment during childbirth with women’s self-report: a validation study in Ghana, Guinea and Nigeria
Source: BMJ Glob Health. 2023 Jul 21;5(Suppl 2):e012122. doi: 10.1136/bmjgh-2023-012122 (PMC10366988; doi:10.1136/bmjgh-2023-012122)
Supplement: Supplementary data [file bmjgh-2023-012122supp001.pdf]

Supplementary file

Supplementary file 1

Reflexivity Statement

1. How does this study address local research and policy priorities?

The overall study was designed as a research collaboration to address the pressing issue of the mistreatment of women during childbirth, which was identified in the study countries (Nigeria, Ghana, Guinea, Myanmar) as a pressing issue affecting maternal health. This study started as a 3-country study (Nigeria, Ghana and Myanmar) and the team from Guinea (CERREGUI) joined our collaboration several months into the planning stage, after they submitted a similar project to the Human Reproduction Programme (HRP) Alliance for Research Capacity Strengthening as part of their long-term institutional development programme plan. For this particular analysis and paper the study team identified and developed this as priority research and policy in their setting.

2. How were local researchers involved in study design?

The overall study design was co-developed through consultation with the whole study group. At an initial project meeting at WHO in 2014, the country research teams (KAB – Ghana, MDB – Guinea, TAI, AKA, and OA- Nigeria) brainstormed appropriate study designs based on their context and in consultation with the WHO research team (OT, MAB). We note with sadness that Prof Bukola Fawole (Nigeria principal investigator) passed away during the project, but participated in these design meetings and led the Nigeria team throughout data collection. HM and HL drafted initial analysis plan. HM led the analysis with support from HL, OT, and MAB.

3. How has funding been used to support the local research team?

At the start of the overall study, we developed a research capacity strengthening plan with the local research teams and WHO researchers, with support from the HRP Alliance for Research Capacity Strengthening. During the project, WHO research team (MAB, JPV, OT) co-facilitated two week-long workshops on data analysis (1-Accra, Ghana 2015, with >20 members of the Nigeria, Ghana and Guinea research teams including KAB, MDB and others not named on this particular paper, 2-Yangon, Myanmar 2015, with 14 members of the Myanmar research team and other junior researchers from the Department of Medical Research) and three week-long workshops on scientific writing (1-Conakry, Guinea 2016, 2-Yangon, Myanmar 2016, 3-Melbourne, Australia 2019). Funding has also been used to facilitate post-study dissemination workshops in Guinea, where the team led by MDB shared the results and implications from the study with the Ministry of Health, professional associations, WHO-Guinea and other key stakeholders: https://www.who.int/news/item/15-05-2020-research-leads-to-actions-improving-childbirth-in-guinea. The overall study has also contributed to KAB , HM and TMM's PhD dissertations (MAB co-supervised TMM's PhD through Khon Kaen University Thailand, where his PhD scholarship was supported by the HRP Alliance for Research Capacity Strengthening).

4. How are research staff who conducted data collection acknowledged?

The paper is one of 17 papers (7 qualitative from the formative phase, 1 protocol, 1 methodological development, and 8 quantitative from the measurement phase) from our research collaboration. Across these 17 papers, research staff responsible for data collection from all 4 countries have contributed as co-authors on at least 1 paper. Each country research team has led at least 2 papers (1 qualitative, 1 quantitative) with their teams. All other research staff contributing to data

Mehrtash H, *et al. BMJ Glob Health* 2023; 5:e012122. doi: 10.1136/bmjgh-2023-012122

collection have been acknowledged.

5. Do all members of the research partnership have access to study data?
All members of the partnership have access to data and have led analysis of data in this and other papers (see #4).

6. How was data used to develop analytical skills within the partnership?
The research team worked collaboratively on data analysis across the whole study and in this particular paper. Two week-long data analysis workshops and three week-long scientific writing workshops were facilitated as part of the research partnership to strengthen the analytic and writing skills for the research team (see #3). For this paper, HM led the analysis with support from HL, OT and MAB.

7. How have research partners collaborated in interpreting study data?
As mentioned in #3 and #6, all research partners have collaborated to interpret the study data during data analysis and scientific writing workshops held across the project. For each multi-country paper produced from our research partnership, discussions were facilitated to understand and co-develop the implications for research, policy and practice for each country.

8. How were research partners supported to develop writing skills?
The research team writing this paper is a mix of senior, mid, and junior academics and clinicians. OIF, TMM, JPV and MAB have completed their PhDs in the last 7 years, and KAB, AKA and HM are currently working on their PhDs. The authorship team was supported by the senior research team to develop and refine writing skills, including through the facilitation of scientific writing workshops (see #3).

9. How will research products be shared to address local needs?
All papers resulting from our research partnership have been published as open access. We have developed a post-publication dissemination plan within WHO and our respective institutions to share our research widely across our networks. The study tools are available freely in eight languages for other research teams to access.

10. How is the leadership, contribution and ownership of this work by LMIC researchers recognised within the authorship?
We note that the majority of authors are researchers from the study countries (Ghana, Guinea, Myanmar Nigeria).

11. **How have early career researchers across the partnership been included within the authorship team?**
We have included early career researchers and clinician researchers (HM, TAI, AKA,, KAB, TMM, MAB) within the authorship team. They have contributed to the data collection, analysis plan, analysis, and writing. We acknowledge that 2 of 8 of the early career researchers are based in high-income countries, and the remaining 6 are based in the study countries (Nigeria, Ghana, Guinea, Myanmar).

12. How has gender balance been addressed within the authorship?
Six authors are female (HM, MAB, TAI, BB, OT, HL) and five are male (AKA, EM,, KAB, TMM, MDB, ,).

13. How has the project contributed to training of LMIC researchers?
The overall study has also contributed to KAB and TMM's PhD dissertations (MAB co-supervised TMM's PhD through Khon Kaen University Thailand, where his PhD scholarship was supported by the HRP Alliance for Research Capacity Strengthening). Please see #3 about analysis and writing workshops facilitated for this project, which were specifically designed by the research teams to address research training needs in each country.

14. How has the project contributed to improvements in local infrastructure?
This project has not directly contributed to improvements in local infrastructure.

15. What safeguarding procedures were used to protect local study participants and researchers?
Adaptations to the study were made based on the local contexts to safeguard our research team. For example, in Myanmar it was not appropriate or safe to conduct labour observations in the maternity ward of public hospitals, so this component of the project was not implemented in Myanmar. Regular debriefing and reflexive discussions between data collectors, country principal investigators and the WHO research team helped to address any issues arising during data collection – this process of reflection during data collection is critical to any research involving sensitive topics or violence to ensure the safeguarding of research participants and research teams.

Supplementary file 2

Content validity and understandability of draft study tools

The development, content validity, and understandability testing of the labour observation and survey tools is described in detail elsewhere (1) . In summary, the draft study tools were first tested with global maternal health technical experts, to evaluate how relevant each item was to the construct it was designed to measure, as well as item clarity, conciseness, feasibility, and any missing items. Then, the survey tool was reviewed by two groups of five women who recently gave birth in Nigeria. The women provided feedback on clarity of wording, understandability, and perceived value of the question to women in their communities. Following revisions to the tools based on these activities, the paper-based version of the tools were piloted in Nigeria by two female researchers. Piloting the labour observation tool was designed to understand potential nuances around inter-rater reliability, identify any barriers or facilitators to implementing the labour observations in busy labour wards, and contribute to development of the study manual of operations. The researchers also piloted the community survey with ten women who recently gave birth to further refine the survey and study implementation. Following the piloting, the paper-based tools were converted into digital forms, with input from the research team responsible for piloting.

Training of data collectors

The data collection training and implementation is described in detail elsewhere (2). Prior to data collection, a training workshop was facilitated for all members of the study team by the principal investigators from each country and WHO. In each country, the data collection training workshops included: (1) an overview of the study and study design; (2) dissemination of results from qualitative formative research; (3) review of the study manual of operations; (4) piloting both paper-based and tablet-based forms; and (5) developing an implementation plan. In order to improve inter-rater reliability particularly in the labor observation tool, training vignettes (Box 1) were developed based on the pre-piloting of tools in Nigeria. These vignettes allowed the data collectors to discuss and practice how to identify and record instances of mistreatment accurately. Following the training workshops, all data collectors piloted both the paper- and tablet-based forms. The labor observation tool was piloted in a hospital that was not part of the study, while the community survey tool was piloted in community settings among women who had recently given birth.

Box 1. Vignettes for labor observation tool

A woman is in labour and a midwife comes to check on her. The midwife slaps the woman's leg to get her attention.

This incident should be completed and submitted as an incident of physical abuse – slapped.

A woman is in labour and a midwife comes to give a vaginal examination. The woman will not open her legs, so the midwife pinches the woman and says "open your legs! You weren't crying about the pain while you were having sex!". The midwife then proceeds with the vaginal examination.

This incident should be completed and submitted as a co-occurring incident. These forms should be completed and submitted simultaneously: (1) physical abuse-pinched, (2) verbal abuse-"received comments about her sexual activity", and (3) vaginal examination.

References

1. Bohren MA, Vogel JP, Fawole B, Maya ET, Maung TM, Baldé MD, et al. Methodological development of tools to measure how women are treated during facility-based childbirth in four countries: labor observation and community survey. BMC medical research methodology. 2018;18(1):132.
2. Bohren MA, Mehrtash H, Fawole B, Maung TM, Balde MD, Maya E, et al. How women are treated during facility-based childbirth in four countries: a cross-sectional study with labour observations and community-based surveys. Lancet (London, England). 2019.

Supplementary Table 1. Sociodemographic and Obstetric Characteristics (N=1136)

	Linked N (%)	Not linked N (%)
**Country**		
Ghana	779 (27.7)	57 (5.0%)
Guinea	425 (50.7)	219 (19.3%)
Nigeria	332 (21.6)	229 (20.2%)
**Age**		
15-19	196 (12.8)	99 (8.8%)
20-29	756 (49.2)	572 (50.3%)
30+	584 (38.0)	465 (41.0%)
**Marital status***		
Single, divorced, or widowed	174 (11.3)	57 (5.0%)
Married or cohabitating	1360 (88.5)	1079 (94.9)
Unknown	2 (0.1)	0
**Education**		
No education	227 (14.8)	128 (11.3)
Some primary education	156 (10.2)	142 (12.5)
Complete primary education	410 (26.7)	280 (24.7)
Complete secondary education	440 (28.7)	322 (28.4)
Complete post-secondary education	262 (17.1)	255 (22.5)
Vocational, other, or unknown	41 (2.7)	9 (0.79)
**Parity***		
1 (first birth)	952 (61.9)	608 (53.5)
2 or more	584 (38.0)	528 (46.5)
**Mode of birth***		
Vaginal	1356 (88.3)	831 (73.2)
Caesarean section	179 (11.7)	304 (26.8)
Don't know	1 (0.1)	1 (0.1)

*p <0.05

Supplementary Table 2. Comparison of self-reported and observed mistreatment by country*

	Percent Agreement	Inflation factor	True reported prevalence (A+C/total)
**Any Physical Abuse**	**84**	**1.2**	**12.4**
GHA	90.4	1.3	6.5
GUI	82.4	0.9	19.1
NGA	70.2	0.8	17.5
**Any Verbal Abuse**	**68.5**	**1.2**	**32.6**
GHA	65.1	1.0	28.8
GUI	72.7	1.5	24.0
NGA	51.2	1.4	47.3
**Failure to meet professional standards of care**			
**Non-consented care among procedures**			
Caesarean section* (N=144)	**76.4**	**1.3**	**13.9**
GHA	n/a	n/a	n/a
GUI	n/a	n/a	n/a
NGA	n/a	n/a	n/a
Episiotomy[1] (N=105)	**61.9**	**0.7**	**72.4**
GHA	n/a	n/a	n/a
GUI	n/a	n/a	n/a
NGA	52.7	0.6	70.9
Vaginal exams (N=986)	**60.5**	**0.9**	**61.6**
GHA	56	0.8	62.3
GUI	79	0.9	57.7
NGA	65	0.9	63.5
Vaginal exams - Private health information disclosed during exam	**82.9**	**0.7**	**17.3**
GHA	92	0.3	6.3
GUI	88	0.6	12.9
NGA	58	0.9	45.8
**Pain Relief Requested[1]**	**80.5**	**0.4**	**18.3**
Not received (n=50)	80.0	1.0	38.0
**No staff member present when baby born[1]**	**93.8**	**1.9**	**2.2**
**Poor rapport between women and providers**			
**Supportive care**			

Woman had a companion present during labour and childbirth	**66.1**	**0.3**	**35.9**
GHA	57.3	0.2	48.0
GUI	85.6	0.9	10.4
NGA	58.4	0.4	40.1
**Autonomy**			
Woman did not have easy access to water or oral fluids during labour[2] (N=1312)	**63.7**	**0.5**	**39.7**
GHA	59.9	0.3	43.0
GUI	71.2	0.4	30.2
NGA	63.3	0.9	44.3
Woman not told she could mobilise during labour, and did not mobilise during labour	**78.1**	**1.2**	**65.0**
GHA	72.8	0.9	84.6
GUI	80.5	2.4	8.5
NGA	87.3	1.0	91.3

*n/a: cell counts <5 not reported

[1] among vaginal births (N=1312)

Mehrtash H, *et al. BMJ Glob Health* 2023; 5:e012122. doi: 10.1136/bmjgh-2023-012122
